# Expression of Galectin-7 Is Induced in Breast Cancer Cells by Mutant p53

**DOI:** 10.1371/journal.pone.0072468

**Published:** 2013-08-14

**Authors:** Carole G. Campion, Marilyne Labrie, Geneviève Lavoie, Yves St-Pierre

**Affiliations:** Institut national de la recherche scientifique, Institut Armand-Frappier, Laval, Québec, Canada; University of Illinois at Chicago, United States of America

## Abstract

Galectin-7 was initially described as a marker of epithelial differentiation expressed in the stratified epithelium of various tissues. Like other members of the galectin family, its expression level is often significantly altered in cancer cells. In breast cancer, its expression is significantly augmented in aggressive molecular subtypes, most notably in estrogen receptor-negative tumors and in cell lines with a basal-like phenotype. Studies using experimental mouse models have further shown high expression of galectin-7 was sufficient to increase the metastatic behavior of poorly metastatic breast cancer cells, rendering them more resistant to apoptosis. This expression pattern in breast cancer cells is unexpected because galectin-7 was originally identified as a *p53*-induced gene. To address this paradox, we have examined the molecular mechanisms regulating galectin-7 in breast cancer cells. Our results showed that transfection of breast cancer cells with expression vectors encoding mutant p53 was sufficient to induce galectin-7 at both mRNA and protein levels. Doxorubicin treatment of breast cancer cells harboring a mutant p53 also induced galectin-7. This induction was specific since knockdown of endogenous mutant p53 inhibited doxorubicin-induced galectin-7 expression. The p53-induced galectin-7 expression in breast cancer cells correlated with increased NF-κB activity and was inhibited by NF-κB inhibitors, indicating that the ability of mutant p53 to induce galectin-7 was dependent on NF-κB activity. The implication of NF-κB was further supported by data showing that NF-κB bound to the endogenous galectin-7 promoter and that TNFα-induced *galectin-7* expression was abolished by NF-κB inhibitors. Taken together, our data provide an explanation to the observed high galectin-7 expression levels in cancer cells and suggest that galectin-7 could be part of a common pathway used by mutant p53 to promote cancer progression.

## Introduction

Evidence suggesting that interactions between lectins and their ligands play a major role in different steps of cancer progression has gained the attention of several oncologists [1]. This is particularly true for galectins. Changes in their expression levels correlate with alterations in cancer cell growth, intercellular adhesion, and apoptosis [2–8]. A case in point is galectin-7. In normal tissues, galectin-7 is present in epithelial cells in various tissues [9–11]. Using tissue microarrays constructed from samples obtained from normal breast tissues and breast carcinomas, we previously reported that galectin-7 was expressed at abnormally high levels in tissues collected from patients with a poor prognosis [12]. These results were consistent with the genomic profiling data previously reported by Perou et al. [13], who provided a molecular portrait of 65 surgical specimens of human breast tumors from 42 individuals. Their data revealed that while *galectin-7* transcripts were expressed at low levels in normal breast tissues and mammary epithelial cell lines, they were highly expressed in estrogen receptor (ER)-negative breast cancer and in cell lines with a basal-like phenotype. This abnormally high expression level of galectin-7 is not restricted to breast cancer cells. It is also found in pancreatic cancer cell lines [14], and in esophageal, buccal, and hypopharyngeal squamous cell carcinoma [10,15–17]. Such high levels of galectin-7 in cancer cells are somewhat paradoxical because galectin-7 has generally been considered a pro-apoptotic protein under the control of p53*. Galectin-7* (also called *p53-induced gene 1*) was indeed identified as one of the 14 transcripts induced by ectopic p53 in colon carcinoma cells [18]. In normal epidermal keratinocytes, increase levels of galectin-7 also correlate with p53 stabilization [19]. Yet, galectin-7 is constitutively expressed in cells harboring mutant *p53* [18,19]. In the present work, we have examined this apparent contradiction by investigating the molecular mechanisms controlling galectin-7 expression in human breast cancer cells.

## Material and Methods

### Cell lines and reagents

Breast cancer cell lines were a generous gift from Dr. Peter Siegel (McGill University, Montreal, Qc, Canada) [20]. Immortalized human keratinocytes (HaCaT) were provided by Dr. Thierry Magnaldo (Génétique et physiopathologie des cancers épidermiques, Faculté de Médecine, Nice, France) [19]. MCF-7 cells were originally obtained from the American Type Culture Collection (ATCC). All cell lines were maintained in complete Dulbecco’s modified Eagle’s medium supplemented with 8% (v/v) FCS, 2 mmol/L L-glutamine and 10 mmol/L HEPES buffer. One mmol/L sodium pyruvate was added for maintenance of MCF-7 cells and one mmol/L of non-essential amino acids for HaCaT cells. All cell culture products were purchased from Life Technologies (Burlington, ON, Canada). Doxorubicin, quercetin and parthenolide were purchased from Sigma Chemicals (St. Louis, MO). Recombinant human TNFα was from R&D Systems (Minneaopolis, MN). Caffeic acid phenethyl ester (CAPE) was from Tocris Bioscience (Ellisville, MO).

### Vectors, transfection and luciferase assay

The plasmids encoding the luciferase reporter vector containing p53 (plasmid 219077) or NF-κB (plasmid 219083) were purchased from Stratagene (Mississauga, ON, Canada). The vectors encoding mutant p53 (R175H, plasmid 16436; R273H, plasmid 16439; V143A, plasmid 16435; R249S, plasmid 16438 and R248W, plasmid 16437 [21]) were obtained from Addgene (Cambridge, MA). The vector encoding the wild-type human *p53* gene was purchased from Origene (Burlington, MA). The expression vector encoding human c-Rel was provided by Dr. Nathalie Grandvaux (University of Montréal, St-Luc Hospital, Montreal, Canada). pSuper and pSuper-p53 siRNA vectors (siRNA CTRL and siRNAp53) were kindly provided by Dr. Reuven Agami (The Netherlands Cancer Institute, Amsterdam, Netherlands) [22]. The pCDNA3.1 vector was purchased from Invitrogen (Burlington, ON, Canada). For transfection, cells were plated at equal density 24 h before transfection. Cells were then transfected with the indicated vector(s) using the Lipofectamine 2000 reagent (Invitrogen) according to the manufacturer’s protocol. After transfection, cells were incubated in complete medium at 37° C in 5% CO_2_ for the indicated periods of time and subjected to a dual reporter assay. Luciferase activity was measured using the Luciferase Assay System protocol (Promega, Madison, WI, USA) and a luminometer (Lumat LB 9507, Berthold). β-galactosidase activity was measured using a colorimetric enzyme assay using the Luminescent β-Galactosidase Detection Kit II according to the manufacturer’s instructions (Clontech Laboratories, Mountain View, CA). Luciferase expression levels were normalized to the levels of β-galactosidase expression.

### RNA Isolation and RT-PCR

Total cellular RNA was isolated from cells using the TRIzol reagent (Invitrogen) according to the manufacturer’s instructions. First-strand cDNA was prepared from 2 µg of cellular RNA in a total reaction volume of 20 µL using the reverse transcriptase Omniscript (QIAGEN, Mississauga, ON, Canada). After reverse transcription, human *galectin-7* (gene ID 3963, sense primer: 5’- ACC AAC CCG GTC CCA G -3’ and antisense primer: 5’- GCG GGC TAA CGC TTT ATT TGC -3’), human *galectin-3* (gene ID 3958, sense primer: 5’- ATG GCA GAC AAT TTT TCG CTC C -3’ and antisense primer: 5’- ATG TCA CCA GAA ATT CCC AGT T -3’), human *TP53* (gene ID 7157, sense primer: 5’- CCA GCC AAA GAA GAA ACC A -3’ and antisense primer: 5’- TAT GGC GGG AGG TAG ACT GA -3’), human *p21WAF1* (gene ID 1026, sense primer: 5’- CTG GAG ACT CTC AGG GTC GAA -3’ and antisense primer: 5’- GGA TTA GGG CTT CCT CTT GGA -3’), human *C/EBPβ* (gene ID 1051, sense primer: 5’-GCG ACG AGT ACA AGA TCC -3’ and antisense primer: 5’- AGC TGC TTG AAC AAG TTC C-3’) and *GAPDH* (gene ID 2597, sense primer: 5’- CGG AGT CAA CGG ATT TGG TCG TAT-3’ and antisense primer: 5’-CAG AAG TGG TGG TAC CTC TTC CGA -3’). cDNAs were amplified using the following conditions: 94^°^ C for 3 min, followed by 35 cycles of the following: 94° C for 1 min, 60° C for 1 minute, and 72° C for 1 min, followed by a final extension step at 72° C for 10 min. PCR was performed in a thermal cycler (MJ Research, Watertown, MA). The amplified products were analyzed by electrophoresis using 1.5% agarose gels and SYBR Safe (Life Technologies) staining and UV illumination.

### p53 knockdown

MCF-7 and MDA-MB-231 cells were plated in 6-well plates at 3x10^5^ cells/well before transfected with pSuper and pSuper-p53 siRNA vectors using the Lipofectamine 2000 reagent (Invitrogen) according to the manufacturer’s protocol. Twenty-four hours after transfection, 0.5 µg/ml of doxorubicin was added, and cells were incubated for an additional 24 h before RNA isolation and RT-PCR.

### Western Blot analysis

Cells were solubilized in radioimmunoprecipitation assay (RIPA) lysis buffer (Thermo, Fisher Scientific, Ottawa, ON, Canada) containing a cocktail of protease inhibitors (Roche, Laval, QC, Canada). Equal amounts of proteins (25 µg) were separated on SDS-PAGE and transferred onto nitrocellulose membranes (Bio-Rad Laboratories, Mississauga, ON, Canada). The membranes were first blocked with 5% milk in PBS/0.05% Tween 20 for 1 h and subsequently blotted overnight at 4° C with primary antibodies: rabbit anti-human p53 polyclonal antibody (1:1000; Santa-Cruz Biotechnology, Santa Cruz, CA) and mouse anti-β-actin monoclonal antibody (1:20000; Sigma). Secondary antibodies consisted of horseradish peroxydase conjugated anti-rabbit or anti-mouse (GE Healthcare, Mississauga, ON, Canada). The immunoblots were developed using ECL detection reagent (GE Healthcare).

### ELISA Assay

Human galectin-7 proteins levels were quantified in whole cells using a commercial ELISA kit (R&D Systems) according to the manufacturer’s protocol. Samples were analyzed using a Model 680 microplate reader (Bio-Rad).

### Chromatin immunoprecipitation (ChIP)

ChIP was performed using the EZ-Chromatin-Immunoprecipitation Assay Kit (Millipore, Billerica, MA) according to the manufacturer’s protocol. Briefly, cells were fixed in 1% paraformaldehyde. Nuclei were isolated, sonicated, and pre-cleaned with protein G agarose/salmon sperm DNA. The pre-cleaned chromatin solution was either set aside as input DNA or incubated with anti-NF-κB/p50 (06-886), (Millipore), anti-NF-κB/c-rel (B-6), anti-p53 (DO-1), or anti-RNA polymerase II (05-623B) (Santa-Cruz Biotechnology) on a rotation platform at 4° C overnight. Input DNA and mouse IgG-pulled DNAs were used as controls for all the experiments. After reversal of the cross-linking, DNA was purified from the immune complex and amplified using PCR primers specific for the *galectin-7* promoter region: sense: 5’-GGC ATA GTC CTA GTG GAT CCC-3’ and antisense: 5’-TGT AAT GGA ACA GCG GCC ACAA-3’. The samples were incubated for 3 min at 94^°^ C, followed by 40 cycles as follows: 1 min at 94^°^ C, 1 min at 62^°^ C, and 1 min at 72^°^ C, with a final extension at 72^°^ C for 10 min. The amplified products were analyzed by electrophoresis using 1.5% agarose gels and SYBR Safe (Life Technologies) staining and UV illumination.

### Confocal analysis

MCF-7 cells were cultured onto glass coverslips to semi-confluency. After 24 h, the cells were washed with cold PBS, fixed with 3% paraformaldehyde in PBS and permeabilized with 0.1% Triton X-100 in PBS and blocked with 1% BSA in PBS (PBA) for 30 min. Cells were first incubated overnight at 4° C with a goat anti-human galectin-7 polyclonal antibody (1:100; R&D Systems) or a rabbit anti-human COX-IV antibody (1:500; New England Biolabs, Ipswich, MA) with PBA. After several washes, the cells were incubated with an Alexa Fluor 488-conjugated donkey anti-goat IgG (1:500; Life Technologies) or an Alexa Fluor 568 goat anti-rabbit IgG (1:500; New England Biolabs) for 1 h at room temperature. Samples were mounted using ProLong gold antifade Reagent with 4’-6-diamino-2-phenylindole (DAPI) (Life Technologies) on glass slides and visualized using a Zeiss LSM780 laser scanning microscope (Carl Zeiss Microimaging, Thornwood, NY).

## Results

### Wild-type p53 up-regulates galectin-7 expression in human breast cancer cells

**Figure 1:**
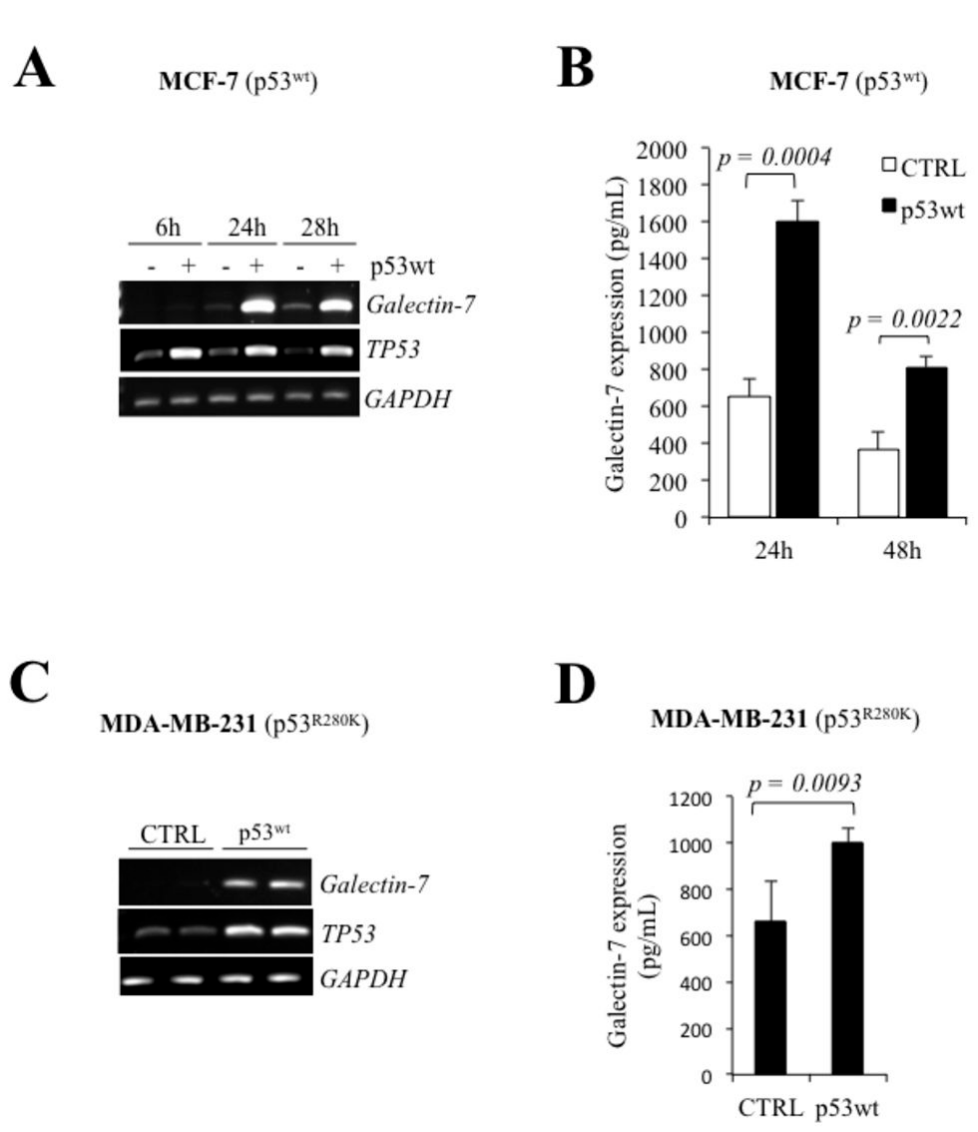
Wild-type p53 induces galectin-7 expression in breast cancer cell. Galectin-7 and *TP53* mRNA and protein levels in MCF-7 (**A** and **B**) and MDA-MB-231 (**C** and **D**) cells following transfection with an expression vector encoding wild type *p53*. Protein levels were measured by ELISA, as described the materiel and methods section. Control cells were generated using an empty pCDNA3.1 vector. *GAPDH* was used as loading control.

**Figure 2:**
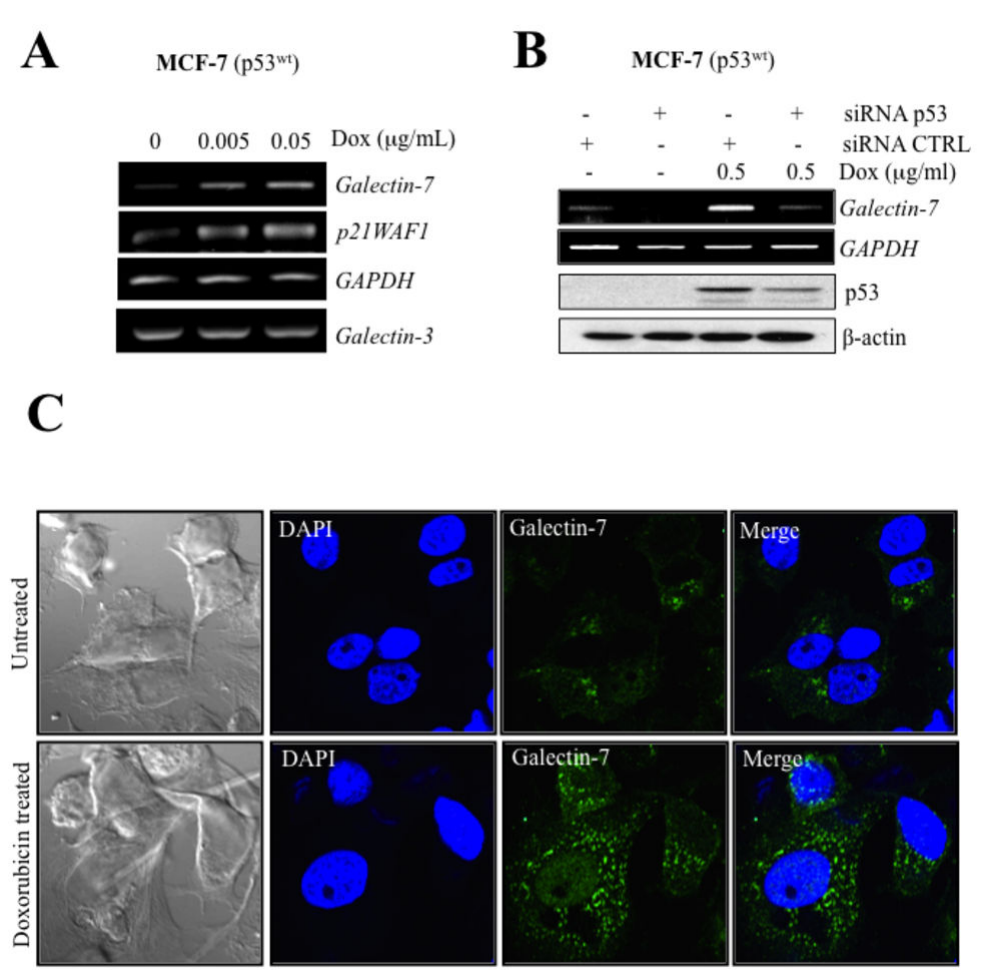
Doxorubicin induces galectin-7 expression in MCF-7^p53wt^. (**A**) Increased expression of *galectin-7* and *p21WAF1* in MCF-7 cells treated with increasing doses of doxorubicin. No such increase was observed in *galectin-3* mRNA levels. *GAPDH* was used as loading control. (**B**) Inhibition of *galectin-7* mRNA levels and p53 protein levels by p53 siRNA in MCF-7 cells treated with 0.5 µg/ml of doxorubicin. A siRNA CTRL vector was used as control and GAPDH or β-actin was used as loading control. (**C**) Confocal images showing galectin-7 expression in doxorubicin-treated MCF-7 as compared to untreated control cells. Fixed cells were labeled with a goat anti-human galectin-7 polyclonal antibody and an Alexa Fluor 488-conjugated donkey anti-goat IgG (green fluorescence). Nuclei were stained with DAPI (blue).

Using MCF-7 cells transfected with a vector encoding wild-type p53 (p53^wt^), we found that increased levels of wild-type p53 were able to induce galectin-7 expression at both mRNA and protein levels (Figure **1A and B**). Similar results were obtained using the MDA-MB-231 cell line (Figure **1B and C**). Treatment of MCF-7 cells, which harbor wild-type alleles of *TP53*, with doxorubicin (Dox) also induced galectin-7 expression at both mRNA and protein levels (Figure **2A and C**). At the protein level, the expression of galectin-7 was restricted to the cytosol and into mitochondria where it colocalized with COX-IV, a well established mitochondrial marker (Figure **S1**). This pattern of expression is consistent with recent data reported [23]. Knockdown of p53^wt^ further inhibited Dox-induced *galectin-7* expression in MCF-7 cells (Figure **2B**)**.**

### Mutant p53 can induce galectin-7 expression in human breast cancer cells

**Figure 3:**
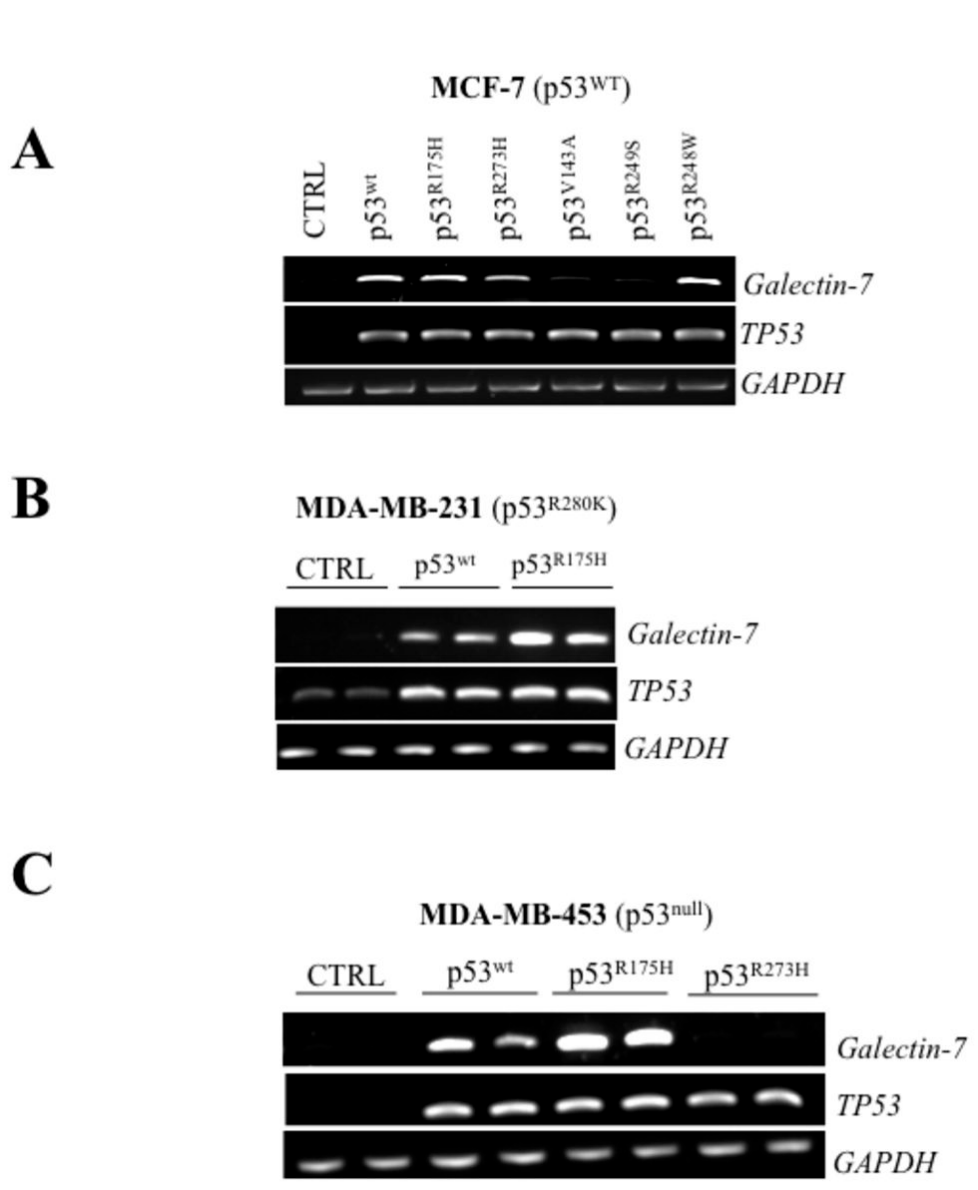
Mutant p53 induces *galectin-7* expression in breast cancer cell lines. Semi-quantitative RT-PCR analysis showing *galectin-7* and *TP53* mRNA levels following transfection with an expression vector encoding the wt p53 or a mutated form of p53 (R175H, R273H, V143A, R249S or R248W) in MCF-7 (**A**), MDA-MB-231 (**B**) and MDA-MB-453 (**C**). An empty pCDNA3.1 vector was used as a control (CTRL). GAPDH is used as loading control.

**Figure 4:**
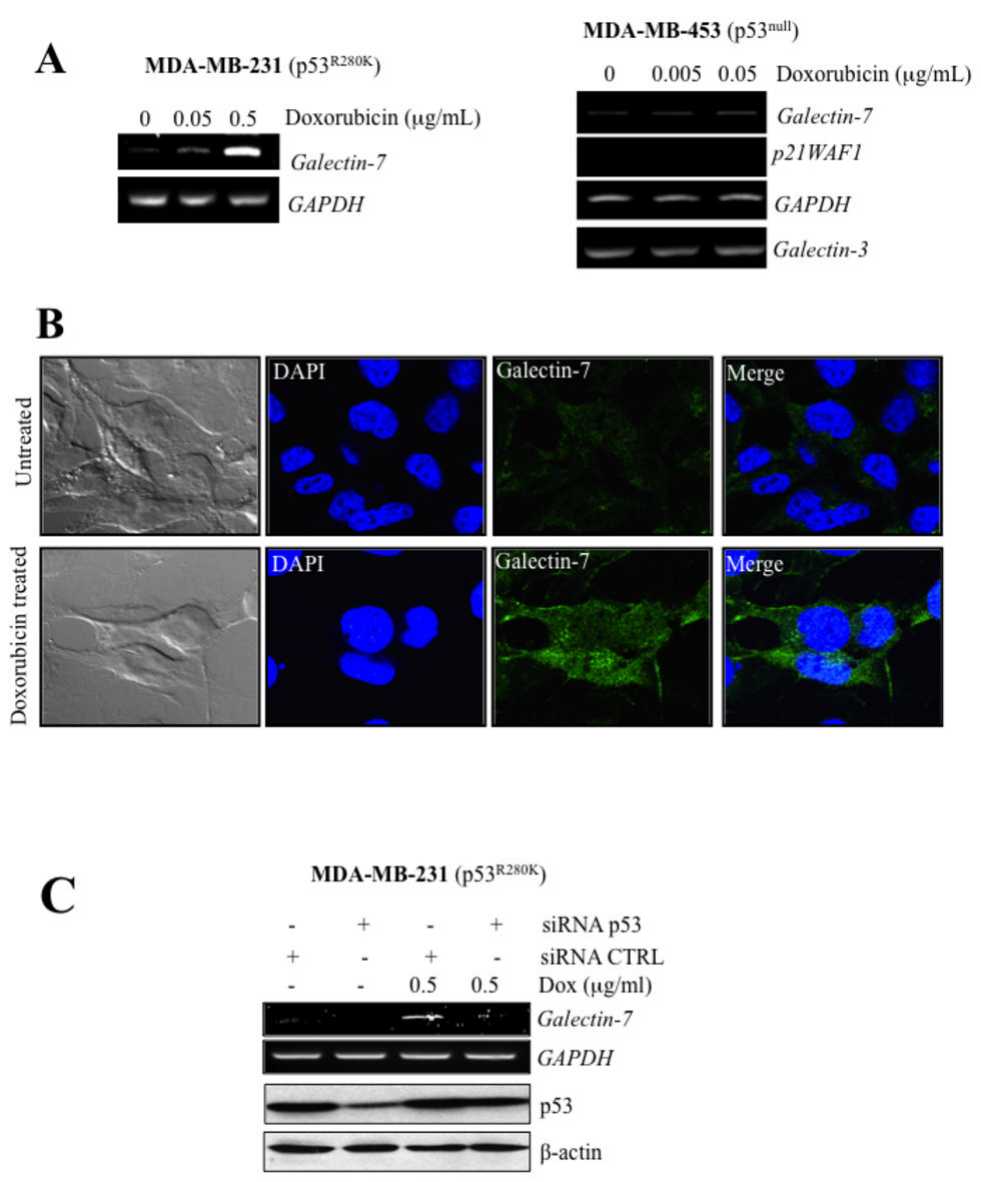
Doxorubicin induces *galectin-7* expression in MDA-MB-231^p53R175H^. (**A**) Effect of increasing doses of doxorubicin on galectin-7 expression in MDA-MB-231, a mutated p53^R280K^ cell line and MDA-MB-453, a p53^null^ cell line. No increase in *galectin-3* mRNA levels was observed in MDA-MB-453. *GAPDH* was used as loading control. (**B**) MDA-MB-231 were treated or not with 0.5 µg/ml of doxorubicin before cells fixation and permeabilization. A goat anti-human galectin-7 polyclonal antibody was used in combination with an Alexa Fluor 488 donkey anti-goat IgG to detect endogenous galectin-7. MDA-MB-231 cells are shown with galectin-7 (green) and nucleus (blue). (**C**) Inhibition of *galectin-7* mRNA levels and p53 protein levels by p53 siRNA in MDA-MB-231 cells treated with 0.5 µg/ml of doxorubicin. A siRNA CTRL vector was used as control and GAPDH or β-actin was used as loading control.

We and others have previously found that galectin-7 was expressed in mammary tissues of aggressive subtypes of breast cancer cells. Because of the high incidence of p53 mutations in human breast cancer, we thus tested whether mutant p53 can also induce galectin-7 expression. To test this, transfected breast cancer cells with vectors encoding the most common forms of mutant p53, including “hot spots” mutants such as p53^R175H^ and p53^R248W^ [24]. Our results showed that p53^R175H^, p53^R273H^ and p53^R248W^ were all able to induce *galectin-7* in MCF-7 (Figure **3A**). No such increase in *galectin-7* was obtained using the control (empty) vector. The p53^R175H^ also induced *galectin-7* when expressed in MDA-MB-231, a human breast cancer cell line harboring a mutant *TP53 gene* (p53^R280K^) and in the p53^null^ MDA-MB-453 cells (Figure **3B, C and S5**). Of note, mutant p53 were not all equally capable of inducing *galectin-7* in all cells types. In MCF-7 cells, for instance, ectopic expression of p53^V143A^ and p53^R249S^ were insufficient to induce *galectin-7*. Similar results were obtained in MDA-MB-453 transfected with a vector encoding p53^R273H^, suggesting that the ability of mutant p53 to induce *galectin-7* was cell-specific. Our results showing that Dox-treated MDA-MB-231 cells expressed increased galectin-7 confirmed that endogenous p53^R280K^ was capable of inducing galectin-7 at both mRNA and protein levels (Figure **4A and B**). No such increase was observed when the p53^null^ MDA-MB-453 cells were treated with Dox, indicating that the effect of Dox was p53-specific. This conclusion is supported by our data showing that Dox-induced *galectin-7* expression by endogenous p53^R280K^ in MDA-MB-231 cells was inhibited by partial knockdown of p53^R280K^ (Figure **4C**).

### The role of NF-κB in galectin-7 expression

**Figure 5:**
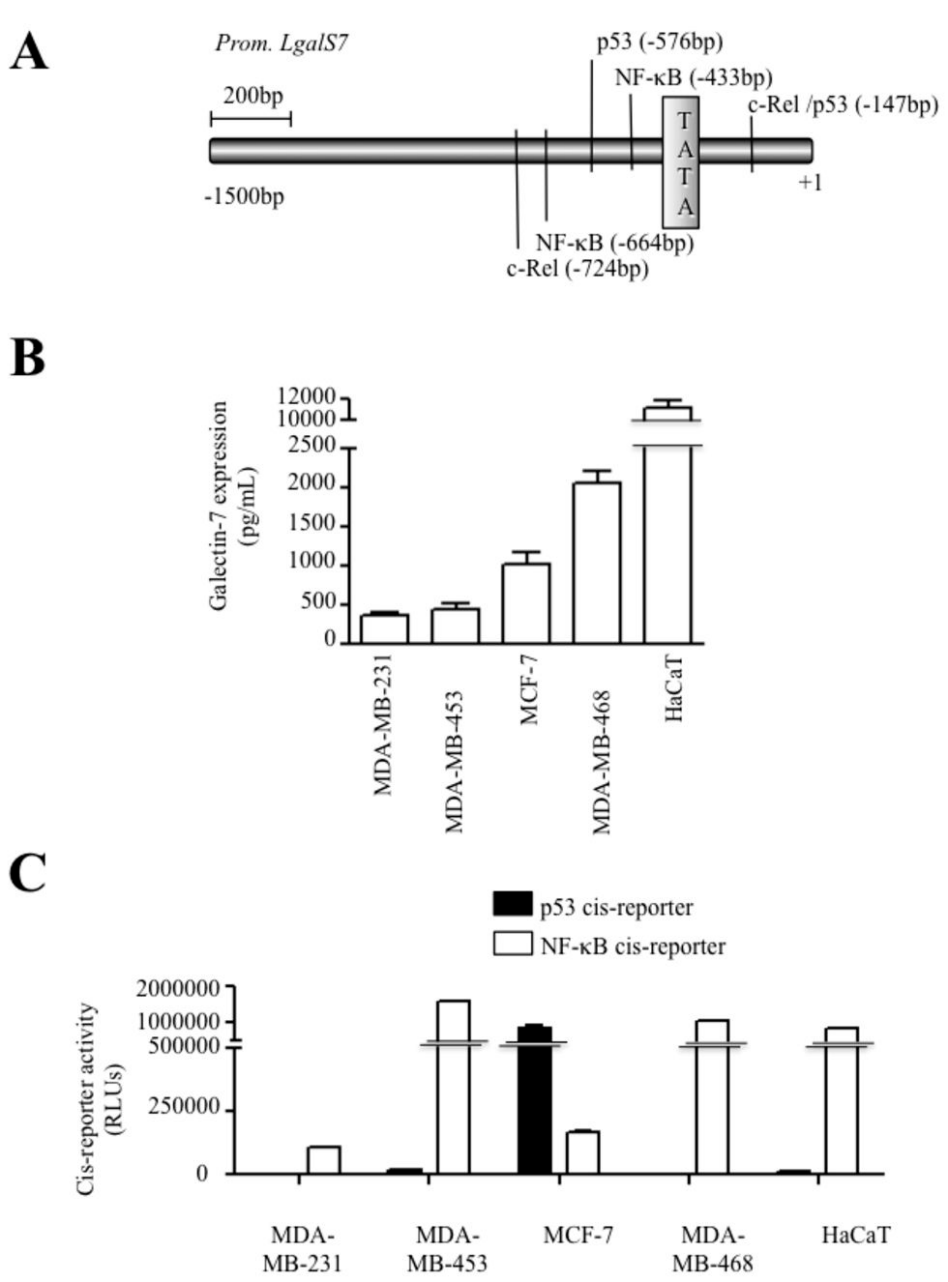
Correlation between galectin-7 expression and NF-κB activity. (**A**) Putative binding sites for NF-κB, c-Rel and p53 within the proximal promoter region of the human *galectin-7* gene. The location of the putative binding sites were obtained by analyzing the sequence of the galectin-7 promoter using the TFSearch program which searches highly correlated sequence fragments against TFMATRIX transcription factor binding site profile database in the 'TRANSFAC' databases by GBF-Braunschweig (http://www.cbrc.jp/research/db/TFSEARCH.html). (**B**) Galectin-7 protein levels measured by ELISA in MDA-MB-231, MDA-MB-453, MCF-7 and MDA-MB-468 breast cancer cell lines. Human epithelial keratinocytes (HaCaT) was used as a positive control. Similar results were obtained by Western blot analysis (**Figure S4**). (**D**) NF-κB and p53 activity as measured following transient transfection with luciferase reporter vectors in breast cancer cell lines with different p53 status and HaCaT cells. Transfection efficiency was normalized with co-transfection with a β-galactosidase reporter vector.

**Figure 6:**
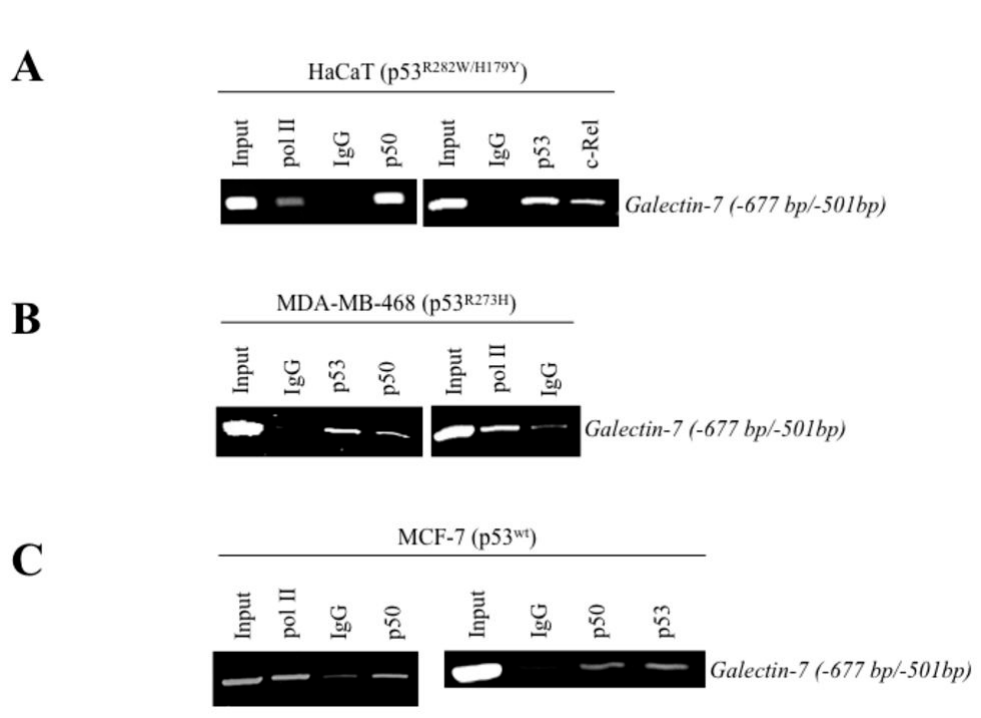
Binding of NF-κB and p53 on the endogenous galectin-7 promoter. Binding of NF-κB p50 and c-Rel isoform, p53 and RNA polymerase II (PolII) on the endogenous galectin-7 promoter was measured by ChIP assay using genomic DNA collected from HaCaT (**A**) and human breast cancer cell lines MDA-MB-468 (**B**) and MCF-7 (**C**). An isotypic control (IgG) was used as a negative control. Total DNA extract was used as a positive control (Input).

**Figure 7:**
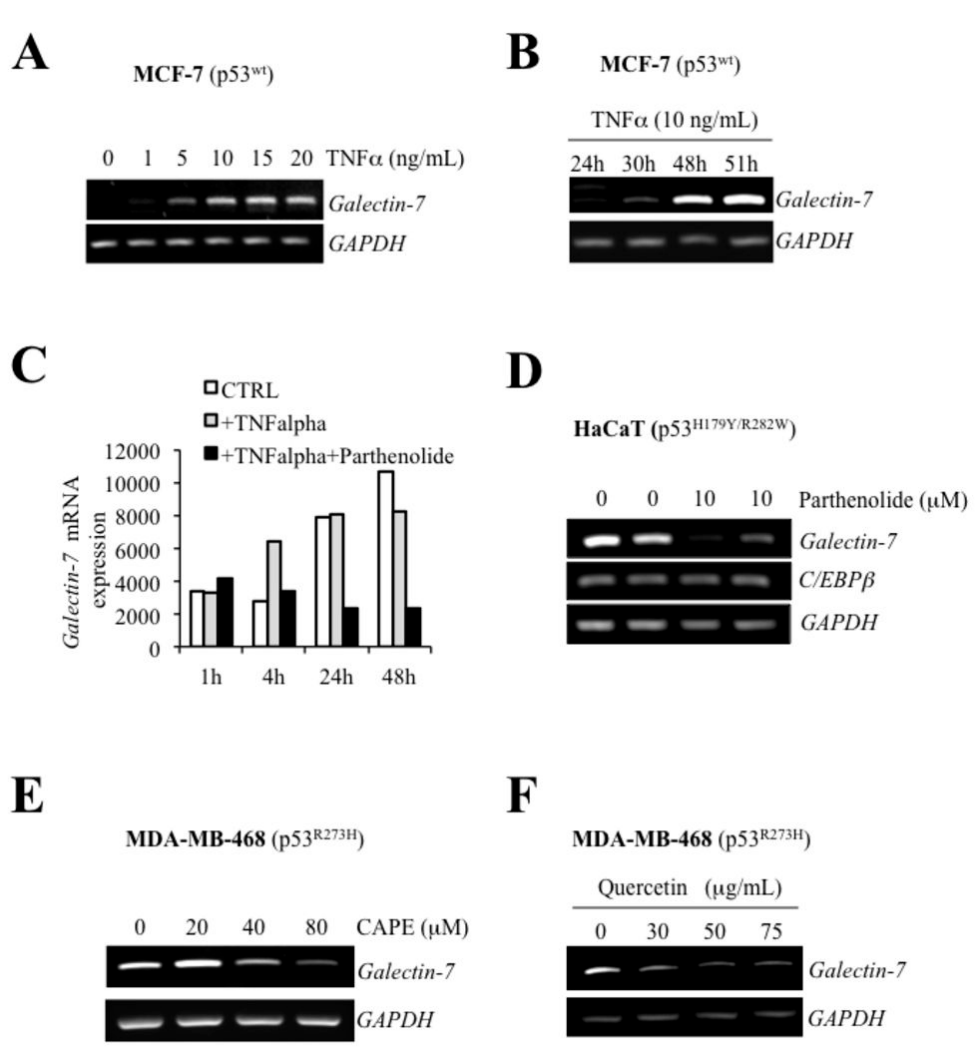
Galectin-7 expression is dependent of NF-κB signalling pathway. mRNA levels following treatment with increasing doses of TNFα (**A**) or at different time post-treatment with TNFα (**B**). (**C**) *In silico* analysis showing effect of parthenolide on TNFα-induced *galectin-7* expression in epidermal keratinocytes. Data were retrieved from a dataset (GDS : 1289) in the Gene Expression Omnibus (GEO) repository. (**D**) Effect of parthenolide on galectin-7 expression in HaCaT cells. mRNA levels of *galectin-7* was measured by semi-quantitative RT-PCR analysis 48h after treatment. No such effect was observed on C/EBPβ expression. GAPDH was used as loading control. (**E**) Dose-dependent inhibition of *galectin-7* mRNA levels in MDA-MB-468 cells treated with CAPE. GAPDH was used as loading control. (**F**) Dose-dependent inhibition of *galectin-7* mRNA levels in MDA-MB-468 cells treated with quercetin. GAPDH was used as loading control.

An *in silico* analysis of the human *galectin-7* promoter revealed the presence of a putative p53 binding site in its 5’ proximal region relative to the transcriptional start site (Figure **5A**). Several putative NF-κB binding sites were also present within this region, raising the possibility that *galectin-7* could be regulated by via a transcriptional crosstalk between NF-κB and p53. This hypothesis is supported by our data showing that both endogenous NF-κB and p53 were equally capable of binding to the human *galectin-7* promoter (Figure **6**). Further support to this hypothesis is provided by our data showing that MDA-MB-468 cells expressing galectin-7 constitutively have high levels of NF-κB activity (Figure **5B and C**). These cells do not have any detectable p53 activity since they have lost the wild-type allele of *TP53* and only express the transcriptionally inactive *TP53* ^R273H^ allele. Similar results were obtained in the case of HaCaT cells, a transformed keratinocyte cell line known to express high levels of galectin-7 [23,25]. Like MDA-MB-468 cells, HaCaT cells do not have detectable p53 transcriptional activity and only express the transcriptionally inactive *TP53* ^H179Y/R282W^ alleles (Figure **5C, S4 and S5**). These results are thus consistent with a role for NF-κB in regulating galectin-7 expression. This conclusion is supported by our data showing that transfection of an expression vector encoding c-Rel or treatment of cells with TNFα a well-known inducer of NF-κB activity, both induced *galectin-7* expression in MCF-7, HaCaT, and MDA-MB-468 (Figure **7A, B and S2**). Our results with TNFα were in fact consistent with gene profiling data in a public profiling database (Geo Profiles, NCBI) showing a specific NF-κB-dependent increase of *galectin-7* in epidermal keratinocytes stimulated by TNFα (Figure **7C**). We also found that parthenolide, a specific inhibitor of NF-κB, suppressed *galectin-7* expression in HaCaT cells (Figure **7D**). Similar results were obtained following treatment of MDA-MB-468 cells with caffeic acid phenethyl ester (CAPE) and quercetin, two other inhibitors of NF-κB [26,27] (Figure **7E and F**). This inhibition of *galectin-7* expression in HaCaT and MDA-MB-468 cells following treatment with parthenolide and CAPE correlated with reduced NF-κB activity (Figure **S3**). Taken together, these results suggest that NF-κB plays a central role in the expression of galectin-7.

### Wild-type and mutant p53 requires NF-κB for the induction of galectin-7.

**Figure 8:**
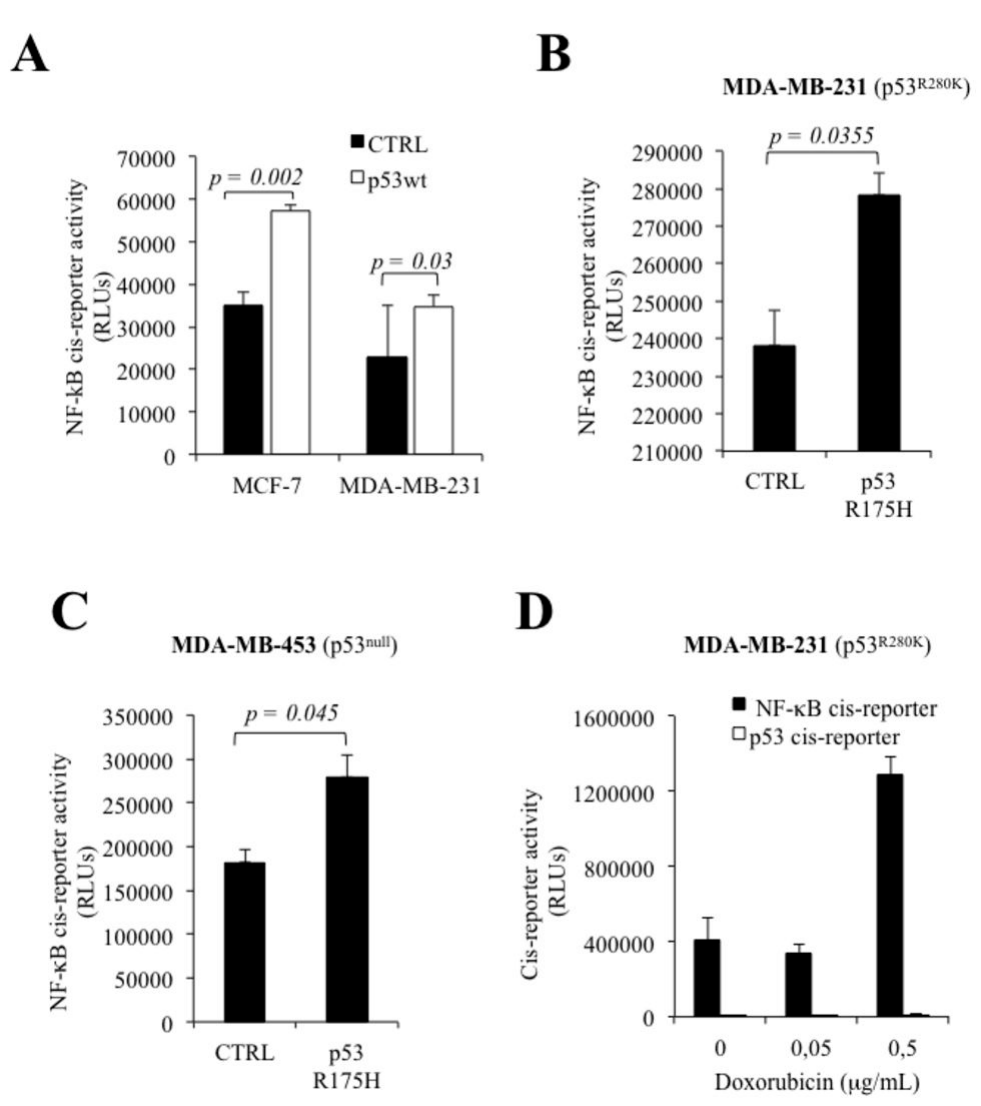
p53 induces galectin-7 expression via NF-κB. (**A**) Increased NF-κB reporter activity in MCF-7 and MDA-MB-231 cells 24h following transfection with an expression vector encoding wt p53. An empty pCDNA3.1 vector was used as a control (CTRL). Transfection efficiency was normalized by co-transfection with a β-galactosidase reporter vector. NF-κB reporter activity in MDA-MB-231 (**B**) and MDA-MB-453 (**C**) cells following transfection of the mutated p53^R175H^. An empty pCDNA3.1 vector was used as a control (CTRL). Transfection efficiency was normalized by co-transfection with a β-galactosidase reporter vector. (**D**) Effect of increasing doses of doxorubicin on NF-κB reporter activity in MDA-MB-231, a mutated p53^R280K^ cell line. Transfection efficiency was normalized by co-transfection with a β-galactosidase reporter vector.

**Figure 9:**
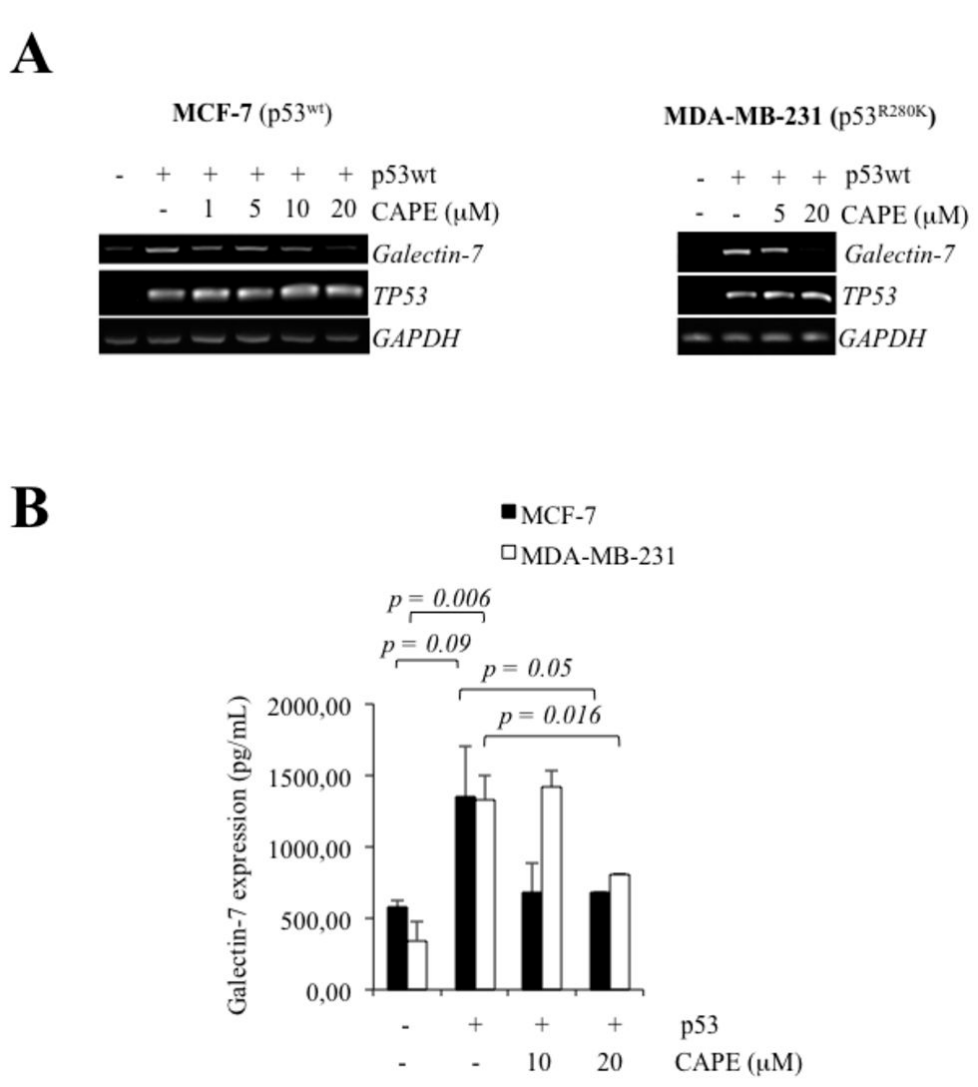
*Galectin-7* expression induced by wild-type p53 required NF-κB. Inhibition of galectin-7 mRNA (**A**) and protein levels (**B**) by increasing doses of CAPE in MCF-7 and MDA-MB-231 cells transfected with an expression vector encoding wt p53. GAPDH was used as loading control.

**Figure 10:**
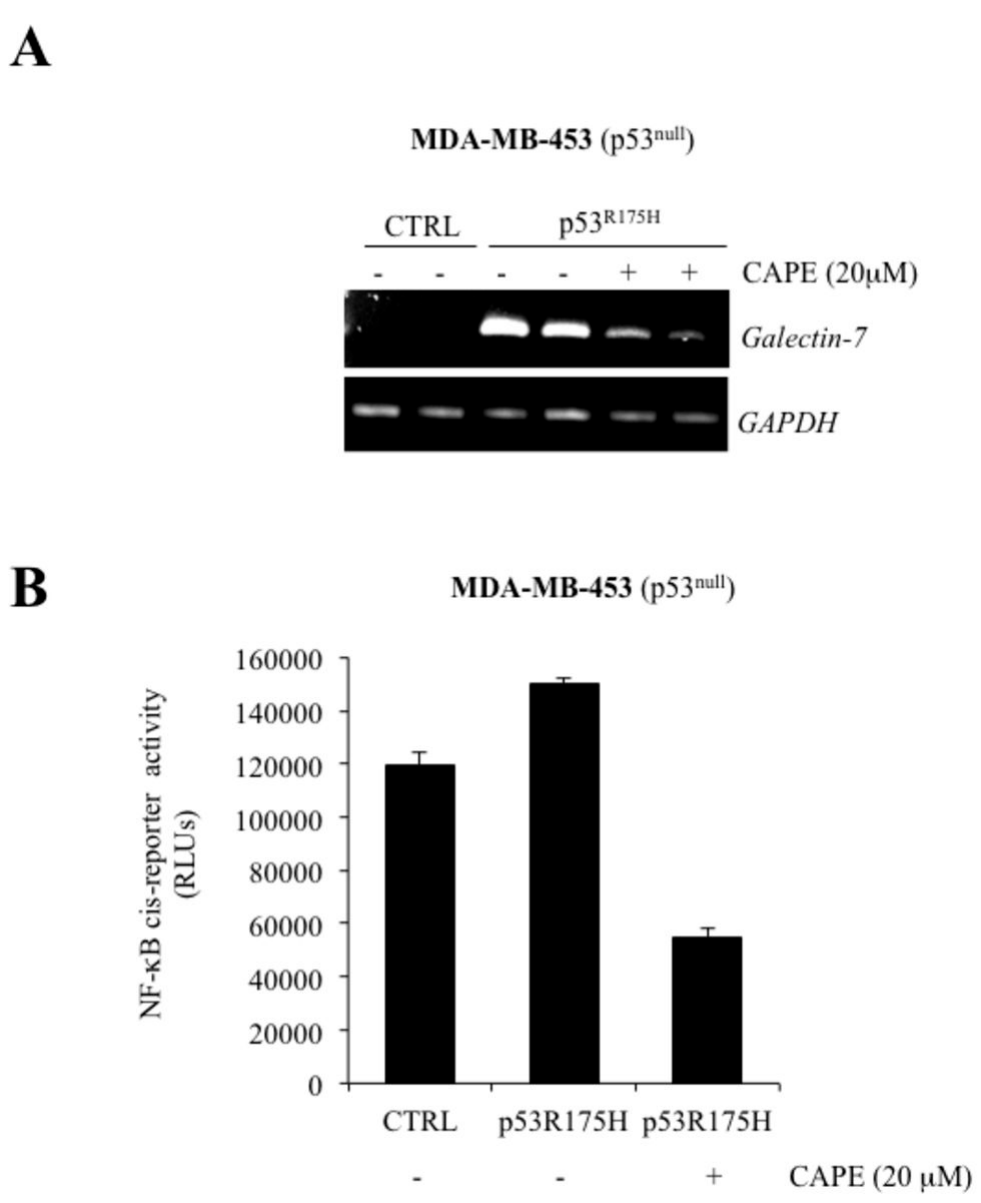
*Galectin-7* expression induced by p53^R175H^ **mutant is inhibited by CAPE**. (**A**) Inhibition of *galectin-7* mRNA levels by CAPE in MDA-MB-453 cells transfected with the expression vector encoding p53^R175H^ or with the (empty) pCDNA3.1 control vector (CTRL). GAPDH was used as loading control. (**B**) Effect of CAPE on NF-κB reporter activity in MDA-MB-453 cells transfected with the expression vector encoding p53^R175H^ or with the (empty) pCDNA3.1 control vector (CTRL). Transfection efficiency was normalized by co-transfection with a β-galactosidase reporter vector.

We next investigated whether NF-κB-dependent galectin-7 expression could be triggered by increased levels of wt or mutant p53. For this purpose, cells expressing wt and mutant p53 were either treated with Dox or transfected with wt or mutant p53. Our results showed that increased expression of wt or mutant p53 (p53^R175H^) both increased NF-κB activity in MCF-7, MDA-MB-231 and MDA-MB-453 cells (Figure **8A–C and S4**). Similar results were obtained using the MDA-MB-231^p53R280K^ cells treated with Dox (Figure **8D and S4**). Moreover, the induction of galectin-7 at the mRNA and protein levels by wt or mutant p53 was inhibited in a dose-dependent manner by CAPE (Figures **9** and **10**).

## Discussion

The present study shows that both wild type and mutant p53 can induce galectin-7 in breast cancer cells. This conclusion is based on the following: 1) transfection with an expression vector encoding wt or mutant p53 induced galectin-7 in several breast cancer cell lines, including p53^null^ cells; 2) treatment of breast cancer cells harboring wild-type or mutated allele of *TP53* with Dox induced galectin-7 expression in human cancer cell lines, and 3) depletion of endogenous p53 inhibited Dox-induced *galectin-7* expression. We further showed that NF-κB was also capable of inducing galectin-7. This conclusion is supported by our data showing that: 1) NF-κB binds to the endogenous galectin-7 promoter; 2) transfection of NF-κB subunits or treatment of cells with TNFα, a well-known inducer of NF-κB, both increased galectin-7; 3) inhibitors of NF-κB decreased constitutive or TNFα-induced *galectin-7* expression. Finally, our results showing that p53-induced galectin-7 expression correlated with increased NF-κB activity and was inhibited by NF-κB inhibitors suggest galectin-7 expression results from a crosstalk between p53 and NF-κB.

Over the last decade, a large number of observations have brought support to the idea that NF-κB plays an important role in cancer. In most cancer cells, NF-κB is constitutively active and is responsible, at least in part, for resistance to apoptosis. It is therefore not surprising that a crosstalk exists between NF-κB and p53. In fact, our results are consistent with a number of studies showing that: 1) mutant p53 expression correlates positively with NF-κB activity in cultured cancer cells [28]; 2) elevated expression of mutant p53 is associated with increased NF-κB activation in cancer tissues [29]; and 3) mutant p53 augments NF-κB activity by affecting both the strength and duration of NF-κB activation [30]. Because high levels of NF-κB have been associated with resistance to apoptosis [31], and galectin-7 induces resistance to apoptosis in breast cancer cells [12], our results suggest that galectin-7 may contribute to NF-κB-mediated resistance to apoptosis. Whether the clinical efficiency of some NF-κB inhibitors alone or in combination with other therapies is linked to reduced galectin-7 is an interesting question that merits future investigations. It will also be interesting to determine whether specific isoforms are all equally capable of binding to the *galectin-7* promoter to induce its expression. Homo- and heterodimerization between NF-κB isoforms generates a diverse range of DNA binding proteins which vary according to the cell type, the stimulus, and the duration of the signals. Using several pharmacological inhibitors to block NF-κB activity thus ensures that we target all NF-κB isoforms. It is important to note, however, there is yet no evidence yet that NF-κB alone is sufficient to induce galectin-7. For example, galectin-7 is not detected in MDA-MB-453 (null p53) cells, which harbor a constitutively high level of NF-κB activity. Moreover, in contrast to other cell models, we could not induce galectin-7 upon treatment of MDA-MB-453 (p53-null) cells with TNFα.

Mutations in the *TP53* gene are among the most common genetic alterations found in breast cancer. The presence of p53 mutations correlates with a high histologic grade, lack of ER and/or PR expression, deregulated apoptosis, and poor prognosis [32]. Such correlation is consistent with the expression pattern of galectin-7, which is exclusively found in ER/PR negative breast cancer [12,13]. Whether galectin-7 is part of a common pathway used by mutant p53 to aid in oncogenesis is an interesting possibility.

The mechanism by which mutant p53 can induce galectin-7 in breast cancer cells via NF-κB is unclear. Given the frequent overlap between p53 and NF-κB binding sites in gene promoters, it is possible that mutant p53 could influence the transcriptional output of specific NF-κB target genes by binding to their promoters. Increased basal NF-κB activity could also result from the ability of mutant p53 to transactivate the *NF-κB2* gene encoding p100/p52 [28]. Perhaps, this NF-κB/p53 complex is required for opening of chromatin at NF-κB loci and for the persistence of NF-κB complex to its binding motifs [33]. It is important to note, however, that a constitutive NF-κB activity is not sufficient to induce galectin-7. For example, the p53^null^ MDA-MB-453 cells do harbor high levels of NF-κB activity but do not express detectable levels of galectin-7 (Figure **5B and C**), suggesting that a NF-κB/p53 complex is required to transactivate the *galectin-7* promoter or that additional epigenetic regulatory mechanisms are involved. Future investigations are needed to clarify this issue. However, our results reveal a regulatory circuit placing NF-κB under the control of p53 and suggest that p53 can dictate cell fate decisions on induction of pro- or anti-apoptotic genes depending on cell status. Cells with p53 levels insufficient or instable to promote transcriptional activities of NF-κB would be disfavored. Inversely, cells with high levels of mutant p53 could mimic NF-κB activation and readily induce expression of anti-apoptotic NF-κB target gene like *galectin-7*. Our results using the HaCaT cell line, a keratinocyte cell line with high levels of NF-κB activity and galectin-7 expression, are interesting in this regard. In these cells, *TP53* is mutated on both alleles, at codon 179 of exon 5 and codon 282 of exon 8 (Figure **S5**). These mutations are commonly found in human tumors and are believed to play a significant role in the immortalization of this line [34]. The mutation at position 179 is known to destabilize the p53 protein by altering its ability to interact with a zinc molecule, causing loss of DNA-binding specificity [33]. While the mutation at position 282 alters the global architecture of the protein, it does not affect its ability to bind to DNA [35]. These specific properties of mutant p53 possibly explain the high transcriptional activity of NF-κB in HaCaT cells and the observed binding of mutant p53 to the endogenous galectin-7 promoter. Similar results were obtained in MDA-MB-468 cells, which express a single *TP53* allele that encodes a missense mutation at codon 273 (R→H) (Figure **S5**). This mutation is one of the most common mutations in cancer cells. It disrupts the sequence-specific DNA-binding core of p53 [36] while maintaining the overall architecture of the DNA binding surface, explaining why binding of specific DNA is not completely abolished. Interestingly, we found that this mutant induced *galectin-7* expression in MCF-7 breast cancer cell line while binding to the endogenous galectin-7 promoter. Surprisingly, it was not able to induce *galectin-7* in the p53^null^ MDA-MB-453 cells. The mutant R249S was also unable to induce *galectin-7* in all cell lines tested, suggesting that cell-specific corepressors/coactivators are involved. We believe, however, that it is too early to determine if a relationship can be established between a specific type of mutation and its effect on galectin-7 expression as we have tested a relatively small repertoire of mutations (Figure **S5**). Moreover, it is important to take into account that the effect of a specific mutation may depend on the cell type and/or the expression of the other wild-type or mutated allele. Future studies will have to be conducted to answer this question. We have nevertheless added a sentence in the discussion regarding this possibility.

In conclusion, we found that both wt and mutant p53 can induce galectin-7 in breast cancer cells. While our data help to explain why galectin-7 is constitutively expressed in cancer cells, they also raise questions about the role of galectin-7 when induced by wt versus mutant p53. Our data support the view that galectin-7 cannot be solely considered a pro-apoptotic protein. The biological role of galectin-7 is likely dependent on functional interactions with other p53-induced genes and/or NF-κB-induced genes, a possibility consistent with its elevated levels in breast cancer tissues where mutant p53 is commonly found. Future investigations will be needed to determine how galectin-7 biological function is modulated by the crosstalk between p53 and NF-κB.

## Supporting Information

Figure S1 (TIF)

Figure S2 (TIF)

Figure S3 (TIF)

Figure S4 (TIF)

Figure S5 (TIF)
