# Supplementary figures and images for: Expression of Galectin-7 Is Induced in Breast Cancer Cells by Mutant p53
Source: PLoS One. 2013 Aug 14;8(8):e72468. doi: 10.1371/journal.pone.0072468 (PMC3743813; doi:10.1371/journal.pone.0072468)

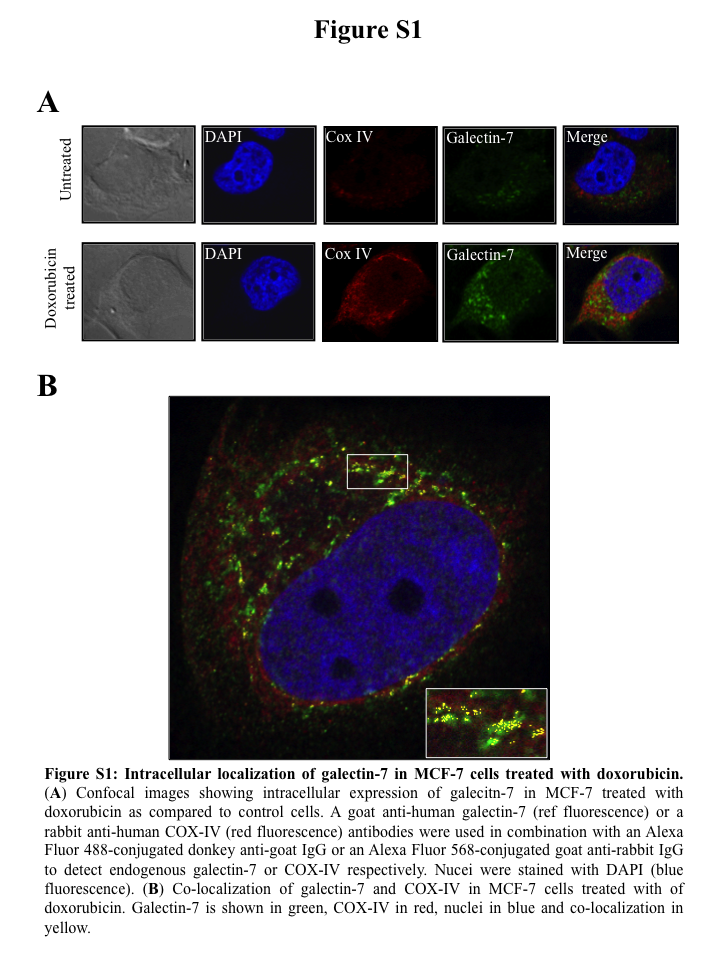

Supplement: Figure S1 — (TIF) [file pone.0072468.s001.tif]

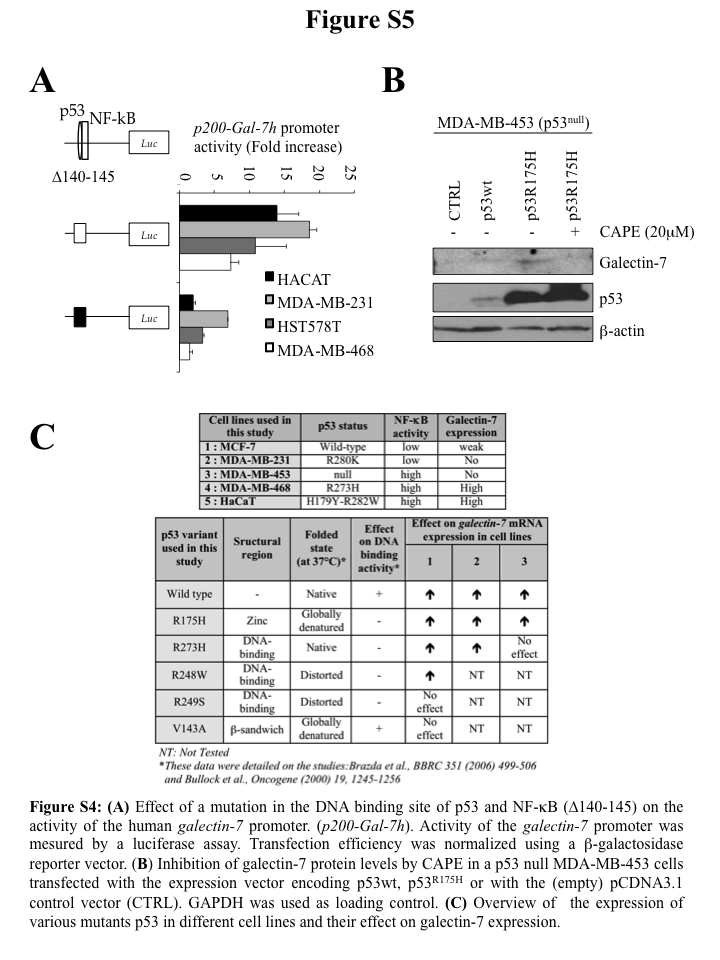

Supplement: Figure S5 — (TIF) [file pone.0072468.s005.tif]
